# Porcine Parvovirus virus-like particle vaccine induces long-term humoral immunity by recruiting T follicular helper and germinal center B cell responses

**DOI:** 10.3389/fimmu.2026.1817304

**Published:** 2026-05-26

**Authors:** Chenyang Hao, Zhenhao Ma, Jiahao Li, Xinxin Qiao, Mengyuan Chen, Jiabin Wang, Yushuo Li, Bei Yao, Jing Zhang, Qun Yang, Pengyu Chen, Dawei Jiang, Pengchao Ji

**Affiliations:** 1 College of Veterinary Medicine, Henan Agricultural University, Zhengzhou, China; 2 International Joint Research Center of National Animal Immunology, Zhengzhou, China; 3 Longhu Laboratory, Zhengzhou, China

**Keywords:** germinal center B cells, humoral immunity, porcine parvovirus, T follicular helper cells, virus-like particles

## Abstract

Porcine parvovirus (PPV) is a major pathogen causing reproductive disorders in sows and remains widely prevalent worldwide. In recent years, PPV has undergone rapid mutations and frequently co-infects with other pathogens, making vaccination the most effective preventive measure. Our previous study showed that co-expression of the chaperone protein Tf16 and VP2 in prokaryotic cells could produce highly immunogenic PPV virus-like particles (VLPs). However, whether these VLP vaccines can induce durable immune memory through sustained antibody recall responses has not been fully elucidated. In this study, mice were immunized with VLPs formulated with ISA 201 VG adjuvant, and the responses of follicular helper T cells (Tfh) and germinal center B cells (GC B cells) were analyzed. The results demonstrated that recombinant VLPs significantly enhanced Tfh and GC B cell responses after the third immunization compared to a commercial inactivated vaccine. More importantly, PPV VLPs effectively induced the generation of specific long-lived plasma cells (LLPCs) and memory B cells (MBCs). These findings enhance our understanding of PPV VLP immunogenicity and may inform the design of more efficacious PPV vaccines.

## 1 Introduction

Porcine parvovirus (PPV) is a major pathogenic agent causing reproductive disorders in pigs (1). Infected sows can replicate and shed the virus without obvious clinical symptoms, while transplacental infection usually results in fetal death and mummification. Once a pig farm is infected, PPV is extremely difficult to eradicate (2). The persistent infection in swine herds, combined with the virus’s high mutation rate, promotes the emergence of new mutant strains (3). To date, vaccination remains the most effective strategy for preventing PPV infection.

The PPV genome is a single-stranded DNA molecule (4, 5), and mature virions have a diameter of approximately 25 nm (6). VP2 is the major capsid protein and determines viral host range and antigenicity; thus, it is widely regarded as the primary immunoprotective antigen in PPV vaccines (7). Notably, VP2 can self-assemble into virus-like particles (VLPs) that mimic the native virion morphology and retain hemagglutination activity (8, 9). As vaccine candidates, PPV VLPs offer a safe and efficacious alternative to inactivated whole-virus vaccines. They elicit robust protective immune responses without the risk of causing disease and provide a versatile platform for delivering epitopes from other pathogens (10, 11).

Protection against PPV relies primarily on humoral immunity. Clinical studies have demonstrated that high levels of neutralizing IgG in pigs effectively control viral infection (12). The germinal center (GC) serves as the key site for antibody affinity maturation and diversification (13). Within GCs, activated B cells undergo proliferation, somatic hypermutation, affinity maturation, and class-switch recombination to produce high-affinity antibodies, making GC formation essential for effective humoral immunity (14). Effective antibody responses require cooperation between B cells and CD4^+^ helper T cells (15). Among CD4^+^ T cell subsets, T follicular helper (Tfh) cells are specifically dedicated to supporting B cell responses (16, 17). Tfh cells regulate the magnitude and quality of GC reactions by providing critical signals, such as IL-21, CD40L, and IL-4, which drive GC B cell expansion, positive selection of high-affinity clones, and differentiation into long-lived plasma cells (LLPCs) and memory B cells (MBCs) (18). Phenotypically, Tfh cells in both mice and humans are characterized by expression of B cell lymphoma 6 (Bcl6) and CXC chemokine receptor 5 (CXCR5), which enable their migration into B cell follicles and localization within GCs (19).

Although numerous studies have explored strategies to induce effective cellular and humoral immunity against PPV (20, 21), the roles of Tfh cells and GC B cells in the germinal center response to PPV vaccination remain largely uninvestigated.

ISA 201 adjuvant is a water-in-oil-in-water (W/O/W) biphasic emulsion that combines favorable safety with immune-enhancing effects (22). Its mechanism of action is primarily reflected in the following aspects: first, it forms an antigen sustained-release system that prolongs the exposure time of the antigen *in vivo*; second, it promotes the uptake and processing of antigens by antigen-presenting cells (APCs), thereby significantly enhancing the intensity of the immune response. Furthermore, ISA 201 adjuvant effectively promotes the GC reaction, enhances the differentiation and expansion of Tfh and GC B cells, thereby improving the level of antibody affinity maturation and contributing to the establishment of long-term immune memory (23).

In this study, the full-length VP2 protein was employed as the antigen construction module, with its core advantages outlined as follows: Preservation of intact conformational epitopes: The full-length VP2 maintains its native tertiary and quaternary structures during VLP assembly, thereby fully retaining conformation-dependent neutralizing antibody recognition sites. Broad epitope coverage: The full-length antigen contains multiple T cell epitopes as well as B cell epitopes, enabling the induction of a broad-spectrum immune response and reducing the risk of immune evasion caused by viral epitope variation (24). VP2 protein is the most important antigenic determinant of PPV, and its immunological characteristics directly determine the protective efficacy of the vaccine. VP2 contains multiple linear and conformational antigenic epitopes, capable of inducing high levels of neutralizing antibodies. The VLPs self-assembled from VP2 are devoid of viral nucleic acids, offering excellent safety. They effectively induce humoral immune responses and a certain degree of cellular immune responses, thereby providing more comprehensive protective immunity (25).

In this study, mice were immunized with recombinant PPV VLPs formulated with ISA 201VG adjuvant. Following the third immunization, Tfh and GC B cell responses in lymph nodes and spleens were evaluated, along with the durability of antibody responses. The results indicate that PPV VLPs induce stronger and more persistent Tfh and GC responses compared to conventional approaches, thereby promoting sustained protective humoral immunity against PPV in mice. These results highlight the potential of PPV VLPs to elicit long-term humoral immunity via enhanced GC reactions, providing valuable insights for the rational design of improved PPV candidate vaccines.

## 2 Materials and methods

### 2.1 Cells, viruses, antibodies, vaccine and animals

PPV-7909 was obtained by the International Research Center for Animal Immunology from the China Institute of Veterinary Drug Control(Bei Jing). Porcine kidney-15 (PK-15) cells (ATCC, USA) were maintained in high-glucose Dulbecco’s modified Eagle’s medium (DMEM; Gibco, USA) supplemented with 10% fetal bovine serum (FBS; Gibco, USA). PPV was propagated in PK-15 cells, and viral titers were determined by 50% tissue culture infectious dose (TCID_50_) assay.

The following antibodies were used for flow cytometry and immunofluorescence analyses (all from BioLegend unless otherwise noted): TruStain FcX™ PLUS (anti-mouse CD16/32) Antibody (clone S17-11E, cat. 156603); APC anti-mouse/human CD45R/B220 Antibody (clone RA3-6B2, cat. 103211); PE anti-mouse CD138 (Syndecan-1) Antibody (clone 281-2, cat. 142503); PE anti-mouse/human CD45R/B220 Antibody (clone RA3-6B2, cat. 103207); FITC anti-mouse/human GL7 Antigen (T and B cell Activation Marker) Antibody (clone GL7, cat. 144603); APC anti-mouse CD95 (Fas) Antibody (clone SA367H8, cat. 152603); FITC anti-mouse CD3 Antibody (clone 17A2, cat. 100203); PerCP/Cyanine5.5 anti-mouse CD4 Antibody (clone RM4-5, cat. 100539); APC anti-mouse CD185 (CXCR5) Antibody (clone L138D7, cat. 145505); PE anti-mouse CD279 (PD-1) Antibody (clone 29F.1A12, cat. 135205); APC anti-mouse CD138 (Syndecan-1) Antibody (clone W20051E, cat. 112507); PerCP anti-mouse/human CD45R/B220 Antibody (clone RA3-6B2, cat. 103233); PE/Cyanine7 anti-mouse CD19 Antibody (clone 6D5, cat. 115519); PerCP anti-mouse IgD Antibody (clone 11-26c.2a, cat. 405735); APC anti-mouse CD38 Antibody (clone 90, cat. 102711); PE anti-mouse/human GL7 Antigen (T and B cell Activation Marker) Antibody (clone GL7, cat. 144607); and LIVE/DEAD™ Fixable Far Red Dead Cell Stain Kit (APC-Cy7; cat. L34973, Thermo Fisher Scientific).

The inactivated porcine parvovirus vaccine (WH-1) has a viral content of ≥10^5.5^ TCID_50_ per milliliter, uses a milky white emulsion as an adjuvant, and The immunization dose for mice is 100 μL (26).

Female BALB/c mice (6 weeks old) were purchased from Liaoning Changsheng Biotechnology Co., Ltd. (Shenyang, China). All animal experiments were conducted in accordance with protocols approved by the Scientific Ethics Committee of Henan Agricultural University. The commercial inactivated PPV vaccine was purchased from Wuhan Keqian Biology Co., Ltd.

### 2.2 Production of PPV VLPs vaccine

Codon-optimized coding sequences of the VP2 protein (GenBank: AAS93264.1 from amino acid 1-579) from PPV (China strain, GenBank: AY583318.1) were synthesized and cloned into the pET28a plasmid. The recombinant plasmid was then transformed into *Escherichia coli* BL21(DE3) competent cells harboring the pTf16 chaperone plasmid, as previously described (26). Protein co-expression was induced with 0.1 mM IPTG and 2 g/L L-arabinose, followed by incubation at 16 °C for 12 h. Following cell disruption, soluble protein expression was evaluated by SDS-PAGE and Western blot analysis. The recombinant VP2 protein was purified by Ni²^+^-NTA affinity chromatography, and its concentration was determined using a BCA Protein Assay Kit (Beyotime).

### 2.3 Identification of PPV VLPs

Cryo-electron microscopy imaging: Purified self-assembled PPV VLPs were sent to Lily Medical Laboratory Center for structural characterization. Transmission electron microscopy (TEM) was performed to visualize the microstructure and self-assembly state of the VLPs.

Hemagglutination titer of PPV VLPs: Hemagglutination assays were conducted in 96-well V-bottom microtiter plates. Briefly, 50 μL of purified PPV VLPs sample was serially diluted two-fold in PBS across the plate, with PBS alone serving as the negative control. Subsequently, 50 μL of 1% (v/v) mouse red blood cell suspension was added to each well. Plates were incubated at room temperature for 20 min, after which hemagglutination patterns were observed. The hemagglutination titer was defined as the reciprocal of the highest dilution of VLPs that resulted in complete agglutination of the 1% v/v mouse red blood cell (RBC) suspension.

### 2.4 BALB/c mouse immunizations

**Figure 1:**
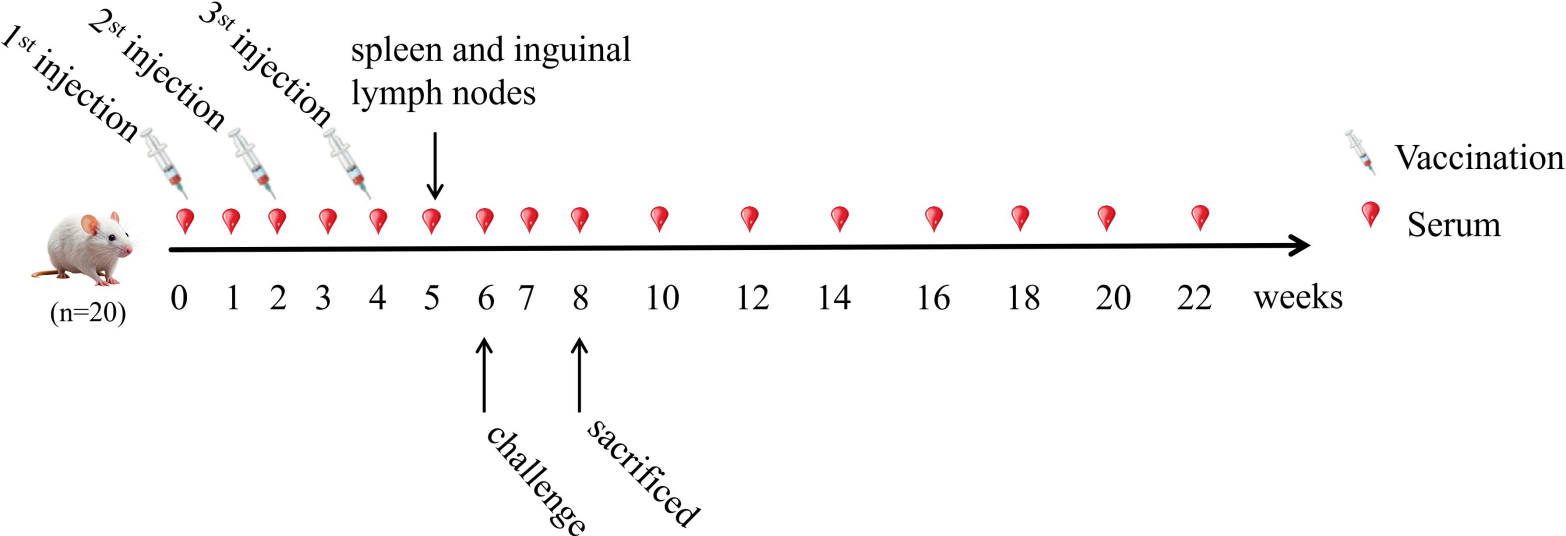
Schematic diagram of mouse immunization schedule and sample collection.

The water-in-oil-in-water (W/O/W) emulsion vaccine was prepared by emulsifying PPV VLPs with Montanide™ ISA 201 VG adjuvant (SEPPIC, Germany) at a 1:1 (mass) ratio. To evaluate the humoral immune response induced by the PPV VLPs vaccine, 6-week-old female BALB/c mice were randomly divided into three groups (n = 20 per group): (1) negative control (100 μL PBS), (2) positive control (100 μL commercial inactivated PPV vaccine), and (3) VLPs group (30 μg VLPs adjuvanted with ISA 201 VG in a total volume of 100 μL). BALB/c mice were immunized via subcutaneous injection in the dorsal region with a volume of 100 μL and an antigen dose of 10^4.5^ TCID_50_.Serum samples(caudal vein) were collected weeklyfrom weeks 0 to 8 post-primary immunization and biweekly from weeks 8 to 22, then stored at -20 °C for future analysis. The immunization schedule was as follows (**Figure 1**):

### 2.5 Detection of serum antibodies

On day 35 post-primary immunization, levels of PPV-specific total IgG and its subclasses (IgG1 and IgG2a) in serum were measured using a commercial indirect ELISA kit for mouse (MultiSciences (Lianke) Biotech Co., Ltd., Hangzhou, China), according to the manufacturer’s instructions. Briefly, test sera were diluted 1:40 in the serum dilution plate, whereas negative and positive controls were diluted 1:4. A volume of 100 μL of each diluted sample (test sera, negative control, or positive control) was added to antigen-coated wells and incubated at 37 °C for 30 min. After three washes with wash buffer, 100 μL of enzyme-conjugated secondary antibody was added to each well and incubated at 37 °C for 30 min. Following another washing step, 100 μL of TMB substrate solution was added per well and incubated in the dark at 25 °C for 10 min. The reaction was terminated by adding 50 μL of stop solution to each well, and absorbance was measured at 450 nm (reference wavelength: 630 nm) within 10 min.

For hemagglutination inhibition (HI) antibody assay, 50 μL of serum was serially diluted twofold in PBS in 96-well V-bottom plates and mixed with an equal volume (50 μL) of 4 hemagglutination units of the PPV 7909 strain. The mixtures were incubated at room temperature for 30 min to allow antibody-antigen binding. Subsequently, 50 μL of 1% (v/v) mouse red blood cell suspension was added to each well, followed by another incubation at room temperature for 30 min. Positive and negative control sera were included in each assay. The HI titer was defined as the reciprocal of the highest serum dilution that completely inhibited hemagglutination of the 1% mouse RBC suspension.

### 2.6 Determination of cytokine levels

On day 35 post-primary immunization, two mice per group were euthanized, and their spleens were aseptically removed. Splenic lymphocytes were isolated and seeded into 12-well plates at a density of 1×10^7^ cells/well, with three replicates per sample. The cells were stimulated with PPV VLP at a concentration of 10 μg/mL. Cell culture supernatants were collected at specific time points post-stimulation: 12 h for TNF-α, 48 h for IL-6, and 72 h for IFN-γ and IL-4. The concentrations of these cytokines were measured using commercial ELISA kits (MultiSciences (Lianke) Biotech Co., Ltd.) according to the manufacturer’s instructions.

### 2.7 Conjugation of VLP with FITC

The VLPs (2 mg/mL) were mixed with FITC (1 mg/mL) at a mass ratio of 1:50 and incubated at room temperature for 8 h with gentle agitation. During the incubation, the reaction tubes were gently inverted every 30 min to ensure thorough mixing and improve labeling efficiency. The reaction was terminated by adding 5 M NH_4_Cl to a final concentration of 50 mM, followed by incubation at 4 °C for 2 h. Unbound free FITC was removed by dialysis (27). The reaction mixture was transferred into a dialysis bag with a molecular weight cutoff (MWCO) of 8 kDa and dialyzed against PBS at 4 °C overnight. The dialysis buffer was replaced 1–2 times during the process to ensure complete removal of free FITC (28). Subsequently, the FITC-labeled VLPs (FITC-VLP) were characterized by spectrophotometric analysis using a microplate reader, and their biological activity was specifically evaluated by a HA assay.

### 2.8 Flow cytometric analysis

To characterize bone marrow plasma cells (BMPCs), mouse bone marrow was harvested on day 56 post-primary immunization and processed into a single-cell suspension. Cells were pre-incubated for 10 min in PBS containing an Fc receptor block and 1% fetal bovine serum (FBS). Cell viability was assessed using the LIVE/DEAD™ (APC-Cy7) Fixable Far Red Dead Cell Stain Kit. Subsequently, cells were stained with surface markers using anti-B220-APC and anti-CD138-PE monoclonal antibodies. Long-lived BMPCs were identified and quantified by flow cytometry. To evaluate the effect of PPV VLPs on the germinal center (GC) reaction and Tfh cell activation, the spleen and inguinal lymph nodes were processed into single-cell suspensions on day 35 post-primary immunization. After Fc receptor blocking and viability staining, GC B cells were identified within the lymphocyte population using a combination of anti-B220-PE, anti-GL7-FITC, and anti-CD95 (FAS)-APC antibodies. Tfh cells were identified using a combination of anti-CD3-FITC, anti-CD4-PerCP-Cy5.5, anti-CXCR5-APC, and anti-PD-1-PE antibodies.

To characterize LLPCs and MBCs, bone marrow and spleen samples were harvested on day 56 post-primary immunization and processed into single-cell suspensions. Following Fc receptor blocking and viability staining, antigen-specific LLPCs were quantified using anti-CD138-APC, anti-CD45R/B220-PerCP, anti-CD19-PE/Cy7, and FITC-labeled VLP. Similarly, antigen-specific MBCs were quantified using a combination of anti-IgD-PerCP, anti-CD19-PE/Cy7, anti-CD38-APC, anti-GL7-PE, and FITC-labeled VLP.

### 2.9 Confocal microscopy

On day 35 post-initial immunization, mouse inguinal lymph nodes were collected and immediately frozen in liquid nitrogen for histological sectioning. Briefly, frozen sections (8 μm) were prepared using a CM1950 cryostat (Leica Microsystems, Germany). The sections were mounted on Superfrost™ Plus microscope slides (Thermo Fisher Scientific, USA), washed with PBS, and fixed with 3% formaldehyde for 15 min at room temperature. Following fixation, the sections were blocked and permeabilized with 2% bovine serum albumin (BSA) for 30 min. For immunofluorescence staining, the sections were incubated with anti-B220-PE and anti-GL7-FITC antibodies (BioLegend, USA) in the dark for 1 h at room temperature. The morphological characteristics and activation status of GCs within the lymph nodes were visualized using a Zeiss LSM700 confocal microscope (Zeiss, Germany).

### 2.10 Real-time quantitative PCR

Total RNA was extracted from lymphocytes isolated from inguinal lymph nodes on day 35 post-primary immunization. The relative mRNA expression levels of key transcription factors and cytokines associated with germinal center (GC) formation and B-cell differentiation, including, *Bcl-2*, *Irf4*, *Prdm1* (Blimp-1), *Pax5*, *Bach2*, and *Il21*, were determined. cDNA was synthesized and analyzed using 2× SYBR Green qPCR Premix (Kermey, China) according to the manufacturer’s instructions. Each sample was analyzed in triplicate. *Gapdh* was used as the internal control, and relative gene expression levels were calculated using the 2^−ΔΔCt^ method. The specific primer sequences are listed in **Table 1**.

**Table 1:** Information of primers used in this study.

Genes	Primer sequence (5’→3’)	GenBank accession No.
GAPDH	F:GGTGGTGAAGCAGGCATCTGAG R:CGGCATCGAAGGTGGAAGAGTG	NM_001289726.2
Pax5	F:CCAGAACAGACCACAGAGTATTCAG R:GGGCTCGTCAAGTTGGCTTTC	NM_008782.3
Bach2	F:ACTGTTGTCGGAGAGGAATCACC R:GCCTGGATCTGCTCTGGACTC	NM_001109661.2
IL-21	F:GTAAAGGGGCACTGTGAGCA R:GGCCACGAGGTCAATGATGA	NM_001291041.1
IRF4	F:CACCTATGATGTTAGCAACCTGGAC R:AGCAACTTCTCAATGTTCTTCCTCTG	NM_001347508.1
Blimp1	F:ATGGTATCAACAACTTCAGCCTCTTC R:GCAGGGAACTCGGTAGGGAAG	NM_001405929.1
Bcl2	F:TGGATGACTGAGTACCTGAACCG R:CAGCCAGGAGAAATCAAACAGAGG	NM_009741.5

### 2.11 Single-cell RNA sequencing

To investigate the transcriptomic profiles of the immune response, inguinal lymph nodes were harvested from mice in the PBS and VLP groups after the third immunization. The samples were immediately processed and submitted to a commercial service provider (NovelBio Bio-Pharm Technology Co., Ltd, China) for single-cell library preparation and sequencing. Single-cell RNA sequencing of mouse inguinal lymph nodes was performed using the 10x Genomics Chromium platform by NovelBio. Approximately 2,0000-3,0000 cells per sample were captured, with an average sequencing depth of 15,000 reads per cell. Raw sequencing data were processed using the CytoNavigator™ single-cell analysis system. Cells with fewer than 200 detected genes, more than 5,000 genes, or mitochondrial gene percentages exceeding 10% were excluded. Gene expression matrices were normalized using the LogNormalize method with a scale factor of 10,000. The top 2,000 highly variable genes were selected for principal component analysis, and the first 20 principal components were used for downstream analysis. Cell clustering was performed using shared nearest neighbor graph-based Louvain clustering with a resolution of 0.5. Subclustering analysis of B and T cell populations was further conducted using a resolution of 0.8. UMAP was used for dimensionality reduction and visualization. A comprehensive bioinformatic analysis was subsequently performed to characterize the enhanced GC B cell and Tfh cell populations induced by PPV VLP vaccination.

### 2.12 Virus challenge and viral load quantification

Previous studies have demonstrated that PPV-7909 is widely used for vaccine efficacy testing and challenge studies (29). On day 42 post-primary immunization, mice were challenged via subcutaneous injection with 100 µL of PPV-7909 virus (10^4.5^ TCID50). Clinical status was monitored daily. At 14 days post-challenge, all mice were euthanized, and spleen tissues were collected for viral load assessment. Total genomic DNA was extracted from the spleens, and the viral load was quantified by RT-PCR. Briefly, DNA samples were normalized to a uniform concentration in TE buffer. A standard plasmid (**Table 2**) containing the *vp2* gene was used to generate a standard curve via 10-fold serial dilutions. Each reaction consisted of 12 µL of PCR master mix and 8 µL of template DNA (sample or standard), performed in triplicate. Nuclease-free water ddH_2_O served as the negative control. The PCR cycling and melt curve analysis were performed on a real-time PCR system. The viral gene copy number was calculated based on the standard curve using the following formula:


$$\mathrm{Copies}/\mathrm{\mu L}=\frac{\left(6.02\times 10^{23}\text{ copies})\times (\mathrm{Plasmid}\text{ concentration}\right)}{\left(\mathrm{Number}\text{ of bases})\times (660\text{ Daltons/bases}\right)}$$

**Table 2:** Primer sequences for the standard plasmid (vp2).

Genes	Primer sequence (5’→3’)
vp2	F:GTGGCGGTGGTCGTGGTG
	R:CGGCTTGCATGTGCGGTAATAC

### 2.13 Statistical analysis

Statistical analysis was performed using GraphPad Prism version 7.0 (GraphPad Software, San Diego, CA, USA). Differences between multiple groups were analyzed using one-way analysis of variance (ANOVA) followed by Tukey’s *post-hoc* test, while comparisons between two groups were performed using Student’s t-test. Data are presented as the mean ± standard error of the mean (SEM). Statistical significance was defined as P < 0.05. Significance is indicated in the figure by asterisks (*, P < 0.05; **, P < 0.01; ***, P < 0.001; ns, not significant).

## 3 Results

### 3.1 Construction and characterization of PPV VLPs

**Figure 2:**
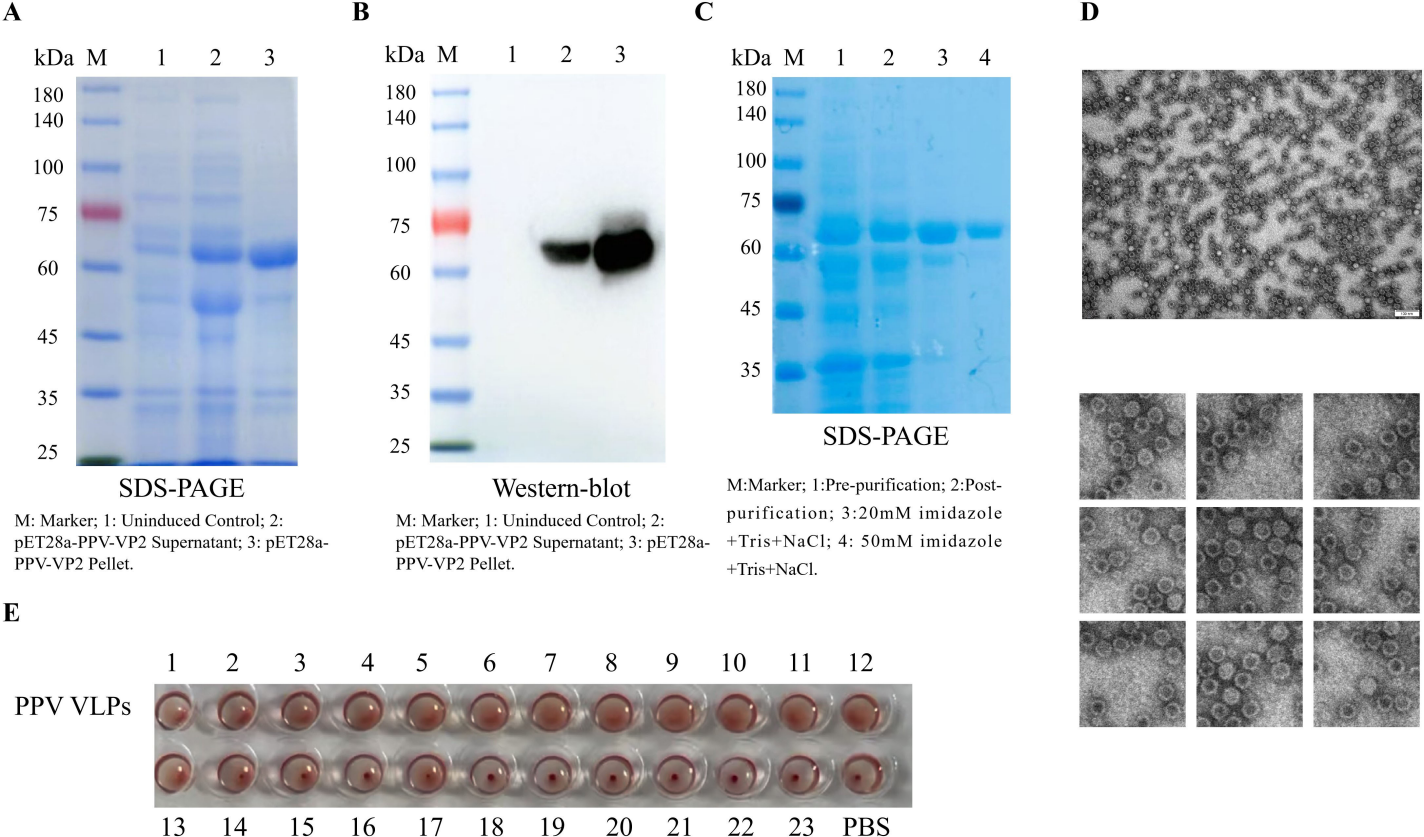
Characterization of PPV VLPs. **(A)** SDS-PAGE analysis of PPV VP2 protein expression. **(B)** Western blot analysis of recombinant VP2 protein. **(C)** SDS-PAGE analysis of purified VP2 protein after Ni²-NTA affinity chromatography. **(D)** Morphological observation of self-assembled PPV VLPs by transmission electron microscopy (TEM). **(E)** Hemagglutination (HA) assay of PPV VLPs. The HA titer was determined using a two-fold serial dilution series; wells 1–23 represent dilutions from 1:2 to 1:2^23^.

Generally, molecular chaperones facilitate protein folding and prevent aggregation, thereby increasing the soluble expression of recombinant proteins in E. coli BL21(DE3) cells (30). In this study, we employed the pTf16 chaperonin system, which significantly enhanced the solubility of PPV VP2 and improved the efficiency of VLP assembly. SDS-PAGE analysis (**Figure 2A**) revealed that the strain co-expressing pTf16 exhibited a significantly stronger soluble protein band at approximately 64 kDa, which was further confirmed as specific PPV VP2 expression by Western blot analysis (**Figure 2B**).

To obtain high-purity PPV VLPs, the supernatant from sonicated BL21(DE3) cells was purified using Ni²-NTA affinity chromatography. The purified VP2 protein demonstrated high yield and purity, appearing as a single band at the expected molecular weight on SDS-PAGE (**Figure 2C**). Following dialysis against Tris-NaCl buffer, the VLP concentration was determined to be 0.66 mg/mL using the BCA assay. Morphological analysis by transmission electron microscopy (TEM) showed that the purified VP2 proteins self-assembled into spherical particles (**Figure 2D**). The biological activity of the self-assembled VLPs was evaluated via a hemagglutination assay. The results demonstrated that the PPV VLPs were capable of agglutinating murine red blood cells, with an hemagglutination titer of 1:2^12^. In contrast, the PBS negative control exhibited no hemagglutination activity (**Figure 2E**). These findings indicate that the self-assembled VLPs possess hemagglutinating functions consistent with those of the native PPV virus.

### 3.2 PPV VLPs stimulate robust antibody responses and CD4^+^ T-cellmediated cytokine production

**Figure 3:**
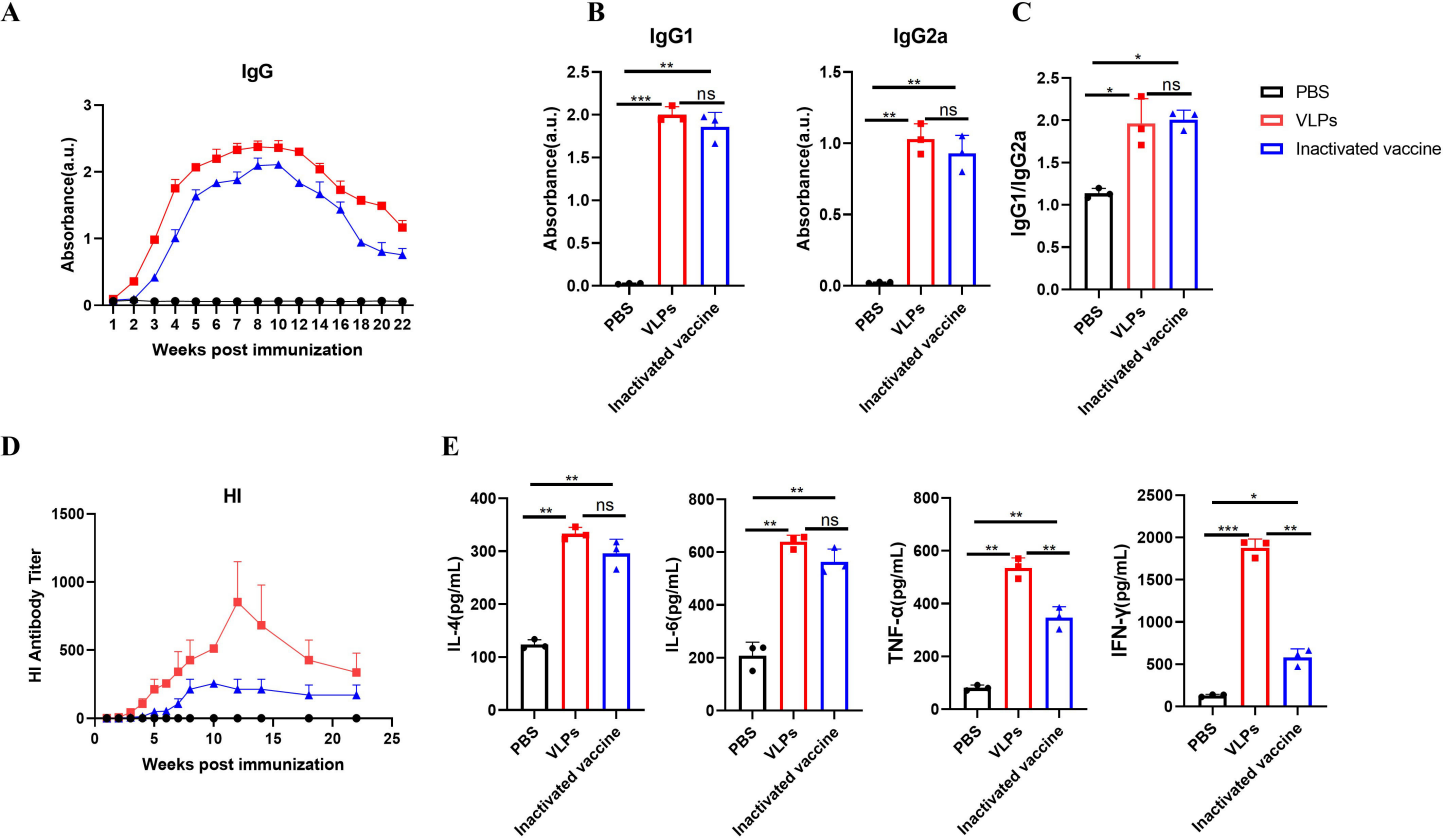
Kinetics and quality of antibody responses and cytokine profiles induced by PPV VLP immunization. **(A)** Kinetic analysis of serum PPV-specific IgG titers. Serum was collected weekly from weeks 1–8 and biweekly from weeks 8-22. **(B)** Levels of IgG subclasses (IgG1 and IgG2a) determined at day 56 post-primary immunization. **(C)** IgG1/IgG2a ratio calculated from day 56 serum samples. **(D)** Kinetic analysis of Hemagglutination Inhibition **(HI)** antibody titers from week 1 to week 22. **(E)** Concentration of cytokines (IL-4, IL-6, TNF-α, and IFN-γ) in splenocyte culture supernatants at day 35 post-primary immunization following antigen restimulation. Significance is indicated in the figure by asterisks (*, P < 0.05; **, P < 0.01; ***, P < 0.001; ns, not significant) (20).

To evaluate the immunogenicity of the PPV VLPs, serum IgG titers and their subclasses were measured following three immunizations. The VLP group exhibited earlier IgG induction compared to the inactivated vaccine group. Throughout the observation period, VLP immunization consistently elicited stronger and more durable IgG responses, maintaining significantly higher titers for up to 22 weeks post-initial immunization (**Figure 3A**). Analysis of IgG subclasses at day 56 revealed that both IgG1 and IgG2a levels in the VLP group were significantly higher than those in the inactivated vaccine group (**Figure 3B**). The IgG1/IgG2a ratios in all immunized groups were all >1, suggesting a Th2-biased immune response that effectively promotes humoral immunity, though no statistically significant difference in this ratio was observed between the VLP and inactivated vaccine groups (**Figure 3C**).

Given that HI titers are typically correlated with neutralizing antibody levels, we monitored HI activity across all groups. Seroconversion was defined as a post-vaccination HI antibody titer ≥8, and seroprotection as a post-vaccination HI antibody titer ≥256 (12). While the PBS control group remained below the detection threshold, PPV VLP immunization elicited a more potent and sustained HI antibody response compared to the inactivated vaccine (**Figure 3D**). HI titers in the VLP group increased rapidly and remained consistently higher than the inactivated group, indicating a superior capacity to induce neutralizing antibodies.

Furthermore, we assessed cytokine secretion by splenocytes to investigate cellular immune responses. Splenocytes from VLP-immunized mice secreted significantly higher levels of IL-4, IL-6, TNF-α, and IFN-γ compared to those from the inactivated vaccine group (**Figure 3E**). Notably, the mean secretion of IFN-γ was approximately three-fold higher in the VLP group. These results demonstrate that while both vaccines can elicit a cellular response, PPV VLPs induce a more robust and balanced Th1/Th2 cytokine profile.

### 3.3 Conjugation of VLP with FITC

**Figure 4:**
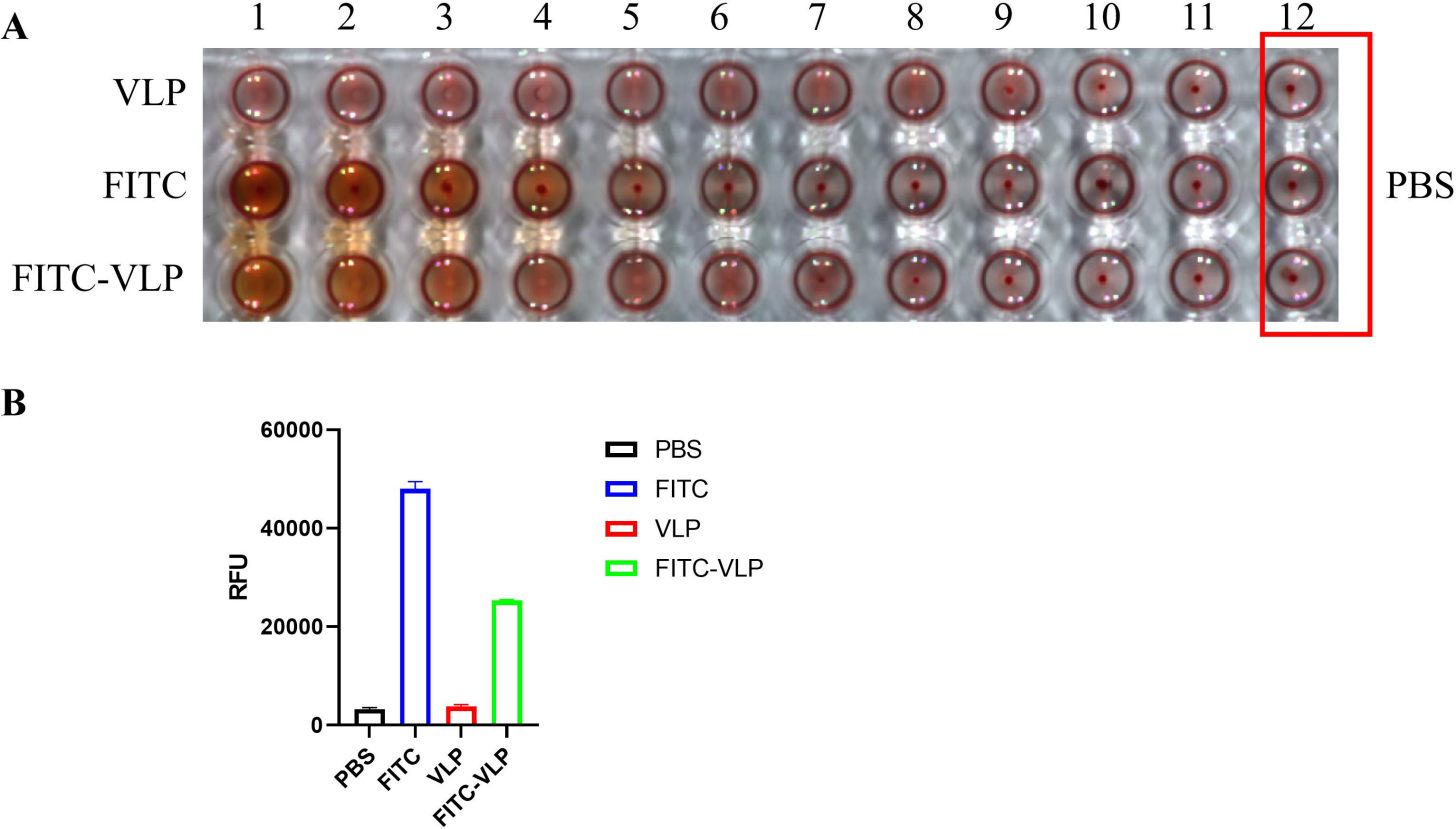
Specific detection of FITC and VLP. **(A)** HA assay was performed on FITC, VLP, and FITC-VLP samples. Well 12 served as the PBS control group. Note: The HA titer was determined using a two-fold serial dilution series; wells 1–11 represent dilutions from 1:2 to 1:2^11^. **(B)** Fluorescence signal intensity of different treatment groups (PBS, FITC, VLP, FITC-VLP) is presented.

The spectrophotometric analysis confirmed (**Figure 4A**) successful conjugation of FITC to VLPs, as indicated by a significant increase in fluorescence signal compared to the unlabeled control. Hemagglutination assay demonstrated (**Figure 4B**) that FITC-VLP retained substantial biological activity, although a slight reduction in HA titer was observed relative to native VLPs, suggesting that FITC labeling did not significantly impair structural integrity or functional properties.

### 3.4 PPV VLPs promote BMPCs response and transcription factor expression

**Figure 5:**
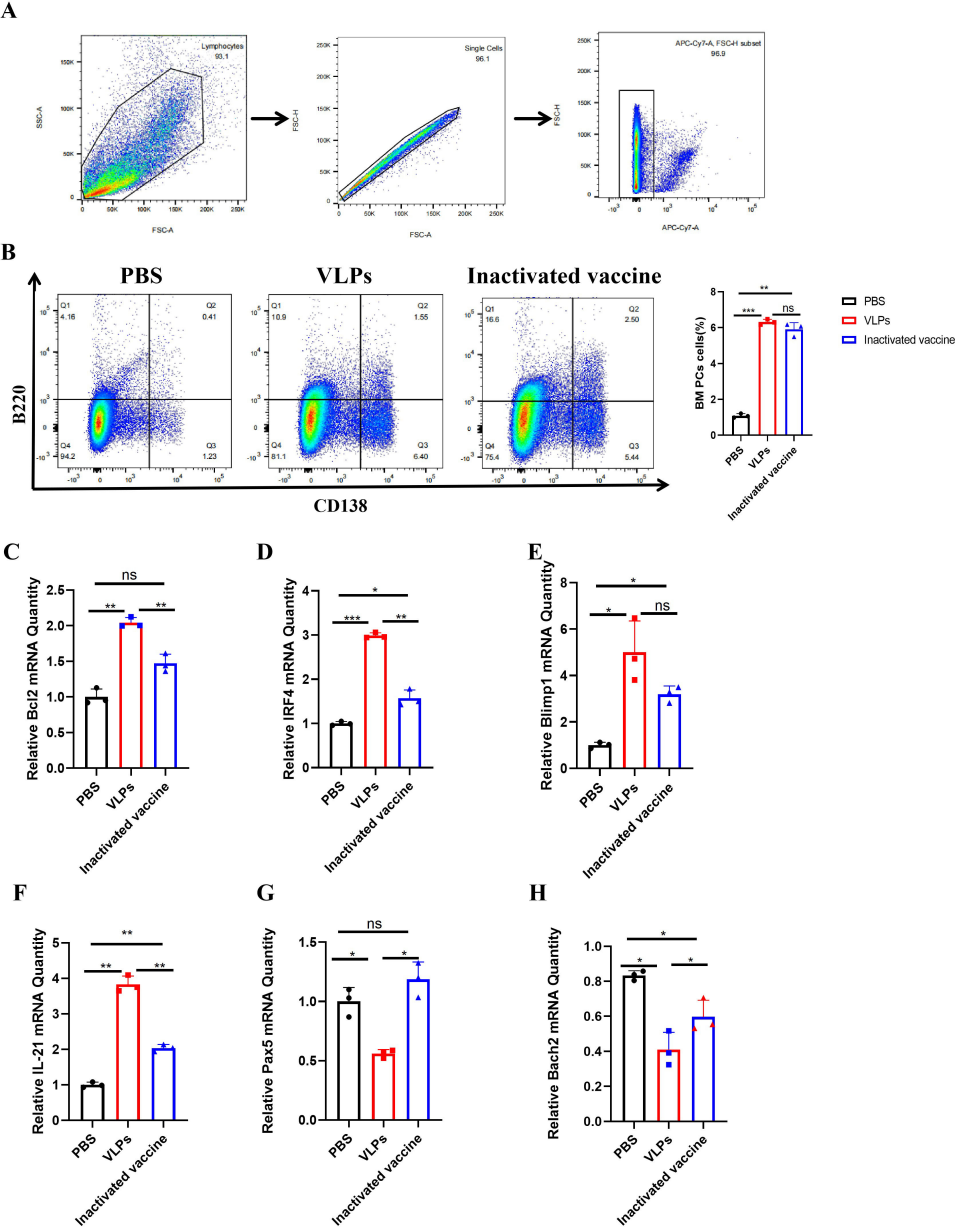
Induction of bone marrow plasma cells and transcriptional profiling in immunized mice. **(A)** Representative flow cytometry gating strategy for identifying BMPCs (B220^-^CD138^+^). **(B)** Statistical analysis of the percentage of BMPCs within total bone marrow cells at 56 days post-primary immunization. **(C–H)** mRNA expression levels of transcription factors and cytokines in inguinal lymph nodes, including *Bcl-2* **(C)**, *Pax5* **(D)**, *Il21* **(E)**, *Bach2* **(F)**, *Irf4* **(G)**, and *Prdm1* **(H)**. Samples for panels A–B were collected at day 56, while **(C–H)** represent data from day 35 post-primary immunization. Significance is indicated in the figure by asterisks (*, P < 0.05; **, P < 0.01; ***, P < 0.001; ns, not significant).

GCs in the lymph nodes are critical sites for B-cell maturation and affinity-based selection, which are essential for the generation of high-affinity antibodies. GC B cells differentiate into memory B cells or plasma cells (PCs), the latter of which are responsible for large-scale antibody production. The gating strategy for BMPCs (B220^-^CD138^+^) is shown in **Figure 5A**. Statistical analysis (**Figure 5B**) showed that, while the inactivated vaccine group induced a detectable proportion of BMPCs, the VLP group exhibited a significantly higher frequency of these long-lived, antibody-secreting cells. This superior response is likely due to the highly repetitive, native-like structure of VLPs, which facilitates efficient recognition and presentation by antigen-presenting cells. When formulated with the potent ISA 201 VG adjuvant, the VLP vaccine significantly enhanced and prolonged B-cell activation, establishing a more robust and durable humoral immune response at the cellular level.

The differentiation of activated B cells into PCs is governed by a coordinated transcriptional program involving several key factors, including *Bcl-2*, *Irf4*, *Prdm1* (Blimp-1), *Pax5*, *Bach2*, and the cytokine *Il21* (31, 32). To elucidate the molecular mechanisms underlying the enhanced BMPC response, we analyzed the mRNA expression levels of these factors in the inguinal lymph nodes via RT-qPCR. The results revealed highly consistent expression patterns: *Bcl-2* (**Figure 5C**), *Irf4* (**Figure 5D**), *Blimp-1* (**Figure 5E**) and *IL-21* (**Figure 5F**) expression in the VLPs group was significantly higher than in the PBS group. *Pax5* (**Figure 5G**) and *Bach2* (**Figure 5H**) showed a coordinated co-expression pattern, with both genes expressed at lower levels in the VLP group compared with the PBS and vaccine groups. These findings indicate that PPV VLPs effectively activate the GC reaction and modulate the transcriptional landscape to favor robust humoral immunity.

### 3.5 PPV VLPs vaccine promote GC B cell activation and Tfh differentiation

**Figure 6:**
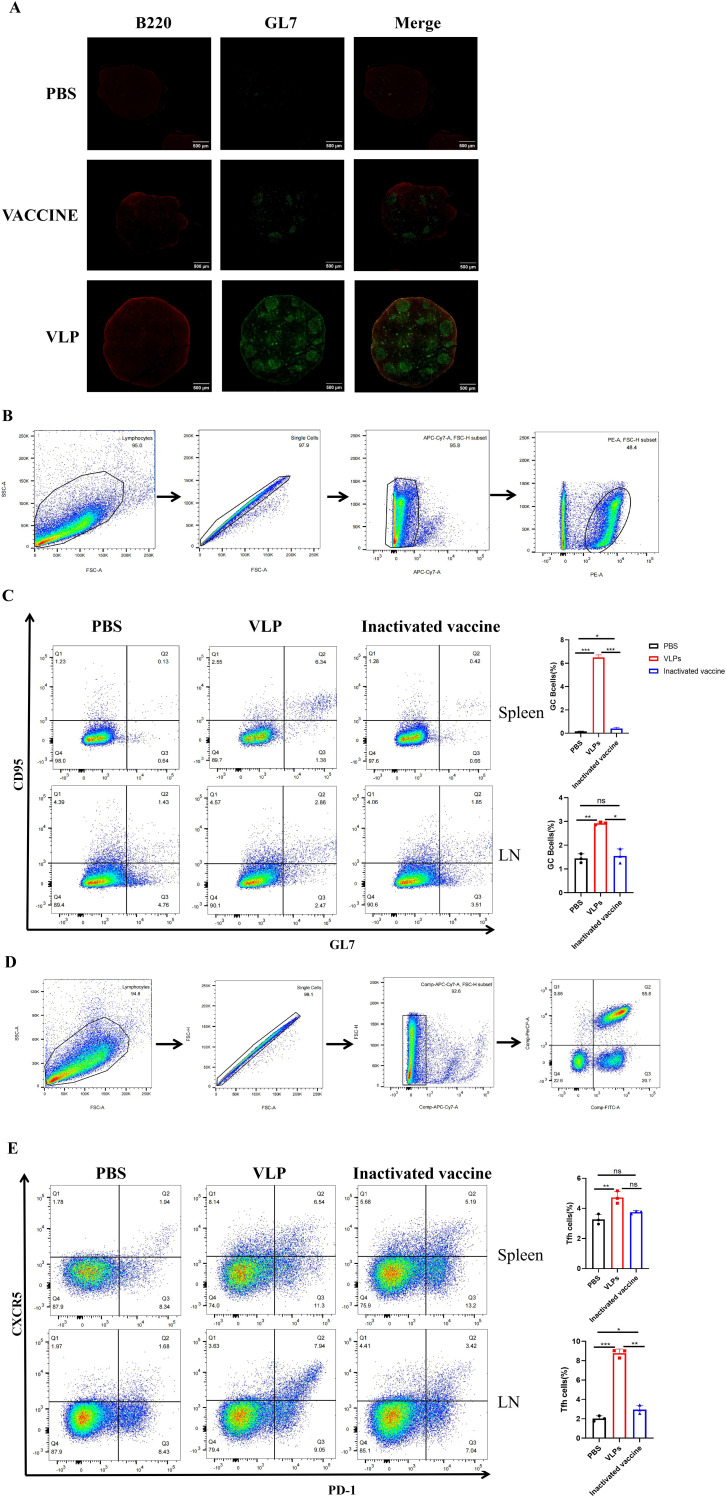
Evaluation of the Impact of PPV VLPs on GC B-cell and Tfh-cell Responses. Spleens and inguinal lymph nodes were harvested from mice on day 35 post-primary immunization. **(A)** Representative confocal immunofluorescence images of lymph node sections stained with anti-GL7 (green) and anti-B220 (red) to visualize germinal center (GC) structures. **(B)** Representative gating strategy for the identification of GC B cells. **(C)** Statistical analysis of the frequency of GC B cells (B220^+^GL7^+^CD95^+^) in the spleen and lymph nodes as determined by flow cytometry. **(D)** Representative gating strategy for the identification of cells. **(E)** Statistical analysis of the frequency of Tfh cells (CXCR5^+^PD-1^+^CD4^+^CD3^+^) in the spleen and lymph nodes. Significance is indicated in the figure by asterisks (*, P < 0.05; **, P < 0.01; ***, P < 0.001; ns, not significant).

GC reactions are primary determinants of the magnitude and quality of antibody responses and humoral memory (33). Given the enhanced B-cell responses observed in the VLP-immunized group, we further investigated the GC reactions using confocal microscopy and flow cytometric analysis. Seven days after the third immunization, frozen lymph node sections were stained for B cells (B220, red) and GC B cells (GL7, green) (**Figure 6A**). Robust GL7^+^GC structures could be easily identified in the group of mice vaccinated with VLP, whereas these structures were sparse in the inactivated vaccine group and absent in the PBS control. These finding suggest that the VLP vaccine recruited a substantial number of B cells into the GCs, facilitating clonal expansion and antibody affinity maturation.

Flow cytometric analysis of lymphocytes isolated from the spleen and lymph nodes (**Figures 6B, C**) provided quantitative data that corroborated these histological observations. The frequency of GC B cells (B220^+^GL7^+^CD95^+^) in the VLP group was significantly higher than that in both the inactivated vaccine and PBS groups. Furthermore, the analysis of Tfh cells, which are crucial for the maintenance and function of GCs, revealed a parallel trend. The proportion of Tfh cells (CXCR5^+^PD-1^+^CD4^+^CD3^+^) in the spleens and lymph nodes (**Figures 6D, E**) of the VLP group was significantly elevated compared to the other groups. Collectively, the high frequency of Tfh cells, the expanded GC B-cell populations, and the upregulated expression of *Il21* mRNA provide a compelling line of evidence that the VLP vaccine successfully activates the GC B cell-Tfh cell axis, the core axis of potent humoral immunity.

### 3.6 PPV VLPs vaccine elicit strong antigen-specific LLPC and MBC responses

**Figure 7:**
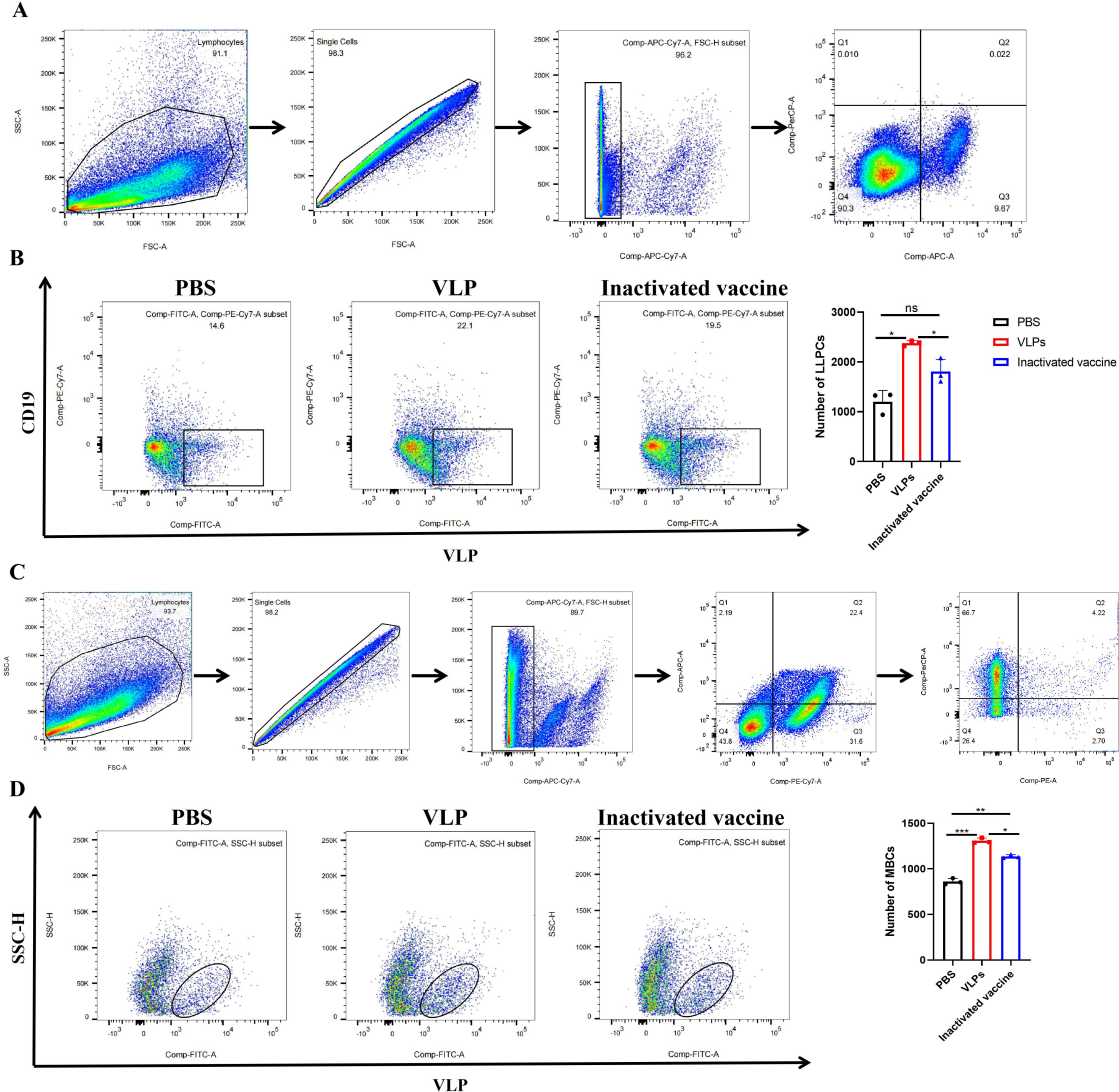
PPV VLPs induce robust antigen-specific LLPC and MBC responses. Bone marrow and spleens were harvested at day 56 post-primary immunization. **(A)** Representative gating strategy for the identification of antigen-specific LLPCs. **(B)** Flow cytometric analysis and quantification of LLPCs (B220^-^CD138^+^CD19^-^) in the bone marrow. **(C)** Representative gating strategy for the identification of antigen-specific MBCs. **(D)** Flow cytometric analysis and quantification of MBCs (CD19^+^ CD38^+^GL7^-^IgD^-^) in the spleen. Antigen-specificity was determined using a VLP-HA fluorescent probe. Significance is indicated in the figure by asterisks (*, P < 0.05; **, P < 0.01; ***, P < 0.001; ns, not significant).

The initiation of GC responses is essential for the development of affinity-matured and durable humoral immunity (33, 34), as it directly drives the generation of LLPCs and memory B cells (MBCs) (18, 35, 36). Given the robust expansion of GC B cells and Tfh cells observed following VLP vaccination, we hypothesized that this formulation would efficiently induce both LLPC and MBC responses. To test this, the absolute numbers of antigen-specific LLPCs (CD138^+^B220^-^CD19^-^VLP ^+^) in the bone marrow and MBCs (IgD^-^CD19^+^CD38^+^GL7^-^VLP ^+^) in the spleen were quantified at week 8 post-primary immunization (**Figure 7**; **Supplementary Figures 1**, **2** shows the FMO controls). Consistent with its superior capacity to promote Tfh and GC B cell formation, the VLP vaccine elicited significantly higher frequencies of VLP-specific LLPCs and MBCs than the inactivated vaccine group. Owing to their highly native-like viral structure, VLPs are more efficiently recognized and processed by antigen-presenting cells (37, 38). When combined with the potent adjuvant ISA 201 VG, the VLP formulation promoted the strongest accumulation of long-lived antibody-secreting plasma cells in the bone marrow niche while simultaneously enhancing the generation of splenic antigen-specific MBCs. This coordinated increase in both LLPCs and MBCs compartments markedly enhanced and prolonged the humoral immune response and was consistent with stronger B cell activation and GC output. At the cellular level, these results confirm that the VLP vaccine induces a more robust and durable humoral immune response characterized by both LLPCs and MBCs dominance.

### 3.7 Single-cell RNA-seq reveals coordinated GC B cell and Tfh cell programs induced by PPV VLP vaccination

**Figure 8:**
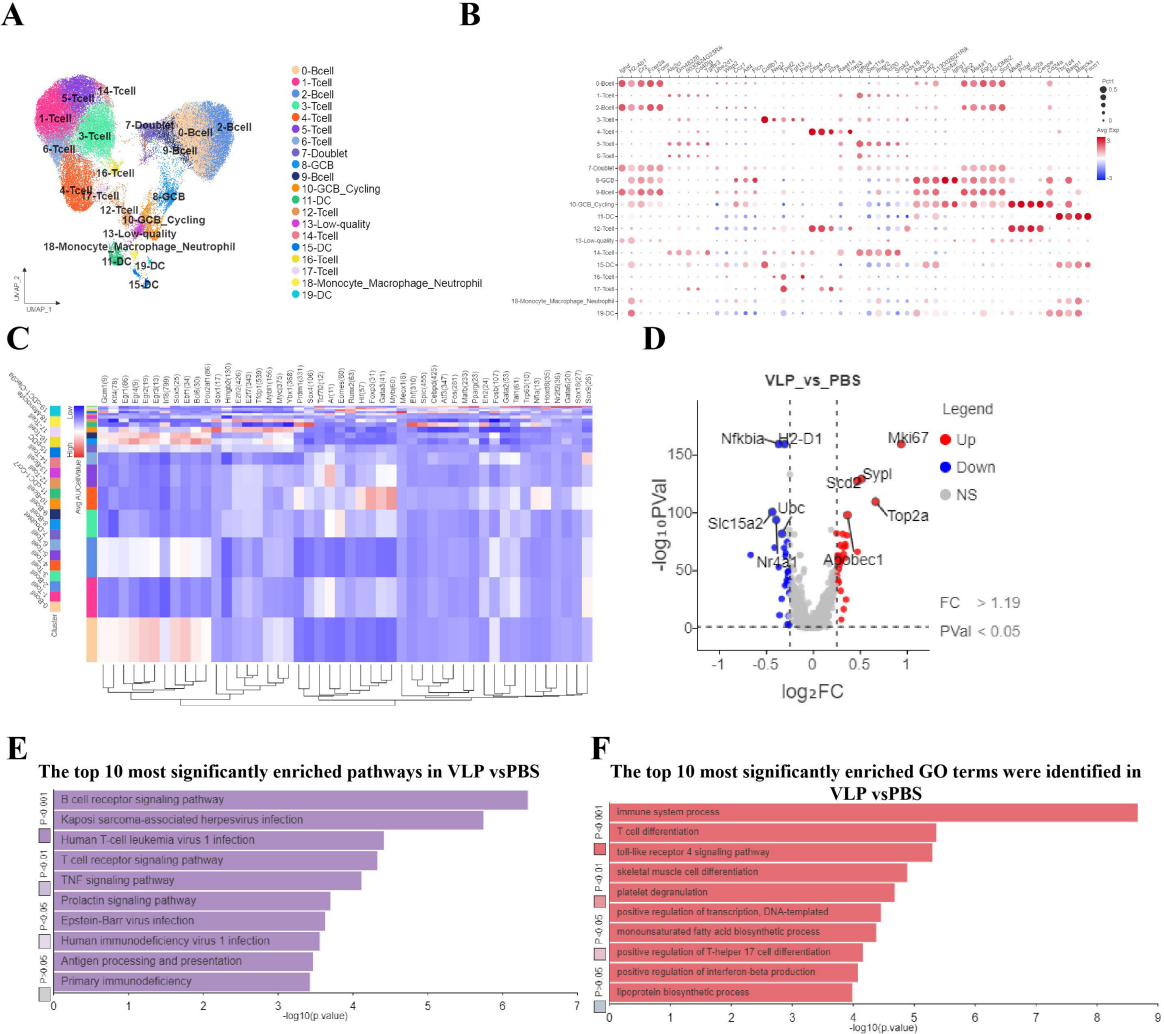
Single-cell transcriptomic landscape of the immune response induced by PPV VLPs. **(A)** UMAP visualization of total immune cells from inguinal lymph nodes, colored by cell type. **(B)** Dot plot showing the expression of canonical marker genes across identified clusters. Dot size represents the percentage of expressing cells; color intensity indicates average expression levels. **(C)** SCENIC analysis showing the activity of key transcription factor regulons across different subsets. **(D)** Volcano plot showing differentially expressed genes (DEGs) in GCB cells between the VLP and PBS groups. **(E)** KEGG pathway enrichment analysis of differentially expressed genes in GC B cells between the VLP and PBS groups. **(F)** Gene Ontology (GO) biological process enrichment analysis of GC B-cell DEGs.

To define the cellular and molecular basis of the enhanced humoral immunity elicited by PPV VLPs, we performed scRNA-seq on lymphoid tissues from VLP-immunized and PBS-treated mice. After stringent quality control and unsupervised clustering, 20 immune cell clusters were resolved and visualized via UMAP, encompassing diverse B-cell and T-cell subsets, PCs, dendritic cells, and myeloid populations (**Figure 8A**). Within the B-cell compartment, we identified transcriptionally distinct populations of naïve B cells, activated B cells, GcB, GCB_Cycling, and PCs. The GCB and GCB_Cycling clusters were characterized by the elevated expression of canonical GC identity and proliferation-associated genes, such as *Bcl11b*, *Lef1*, *Tcf7*, *Igkc*, *Mki67*, *Top2a*, *Pcna*, and *Cenpe*, consistent with active clonal expansion and affinity maturation (**Figure 8B**). In contrast, PC clusters exhibited strong expression of *Ighg1* and other immunoglobulin genes, supporting a robust antibody-secreting phenotype.

Within the T-cell compartment, multiple CD4^+^ subsets were resolved. A distinct cluster expressing *Tcf7*, *Icos*, *Ctla4*, *Cd28*, and *Bcl11b*, but lacking cytotoxic signatures, was identified as Tfh-like cells, specialized for GC support and B-cell help (**Figures 8B, C**). Comparative analysis of cell-type composition demonstrated that PPV VLP immunization significantly increased the relative abundance of B cells and PCs, while selectively enriching GC-associated B-cell subsets compared with PBS controls (**Figure 8A**). In parallel, helper T-cell populations expanded preferentially over cytotoxic subsets, indicating a humoral-biased immune remodeling. These cellular changes were concordant with elevated PPV-specific IgG and hemagglutination-inhibition antibody titers observed at the serological level.

To further define the molecular *programs* underlying GC B cell activation, differential gene expression (DGE) analysis was performed. Volcano plot analysis revealed significant upregulation of proliferation- and activation-associated genes, including *Mki67*, *Top2a*, *Sdc2*, *Sypl*, and *Apobec1*, in the VLP group, whereas inhibitory or quiescence-associated genes such as *Nr4a1* and *Nfkbia* were relatively downregulated (**Figure 8D**). These changes indicate a shift toward an activated and proliferative GC B-cell phenotype following VLP immunization. To gain functional insight into these transcriptional changes, pathway enrichment analysis was conducted. KEGG analysis of GC B differentially expressed genes revealed significant enrichment of immune-related pathways, including the B-cell receptor signaling pathway, T-cell receptor signaling pathway, antigen processing and presentation, and notably the TNF signaling pathway (**Figure 8E**). The emergence of TNF signaling as a top enriched pathway suggests that inflammatory and NF-κB-linked signaling cascades may play a critical role in supporting GC activation and downstream humoral immunity following VLP vaccination. Consistently, Gene Ontology (GO) enrichment analysis demonstrated that VLP-induced transcriptional programs were strongly associated with broad immune activation processes, including immune system process, T-cell differentiation, toll-like receptor 4 signaling, and regulation of interferon-related responses (**Figure 8F**). These results further support that PPV VLP vaccination promotes coordinated immune remodeling within the lymphoid microenvironment, enhancing both GC-associated B-cell activation and helper T-cell functional differentiation.

Collectively, these single-cell transcriptomic and enrichment analyses demonstrate that PPV VLP vaccination promotes the coordinated expansion and activation of the GC-Tfh-plasma cell axis, characterized by enhanced BCR signaling, GC-supportive transcriptional programs, and prominent activation of the TNF signaling pathway. This integrated molecular and cellular network provides mechanistic insight into the improved germinal center responses, enhanced affinity maturation, durable antibody production, and the generation of high-quality humoral immunity elicited by PPV VLPs.

### 3.8 Mice vaccinated with PPV VLPs display a better immunoprotection against virus challenge

To determine whether the enhanced recruitment of Tfh cells, GC B cells, and plasma cells translates into functional immunity, we evaluated the protective efficacy of the PPV VLPs in a virus challenge model. Since PPV infection in mice typically does not result in overt clinical symptoms or significant pathological lesions, protective efficacy was assessed by quantifying the viral load in target tissues. Immunized mice were challenged subcutaneously with the cell-cultured PPV-7909 virus. At 14 days post-challenge, spleens were harvested for total DNA extraction, and the viral burden was quantified by absolute qPCR targeting the *vp2* gene.

**Figure 9:**
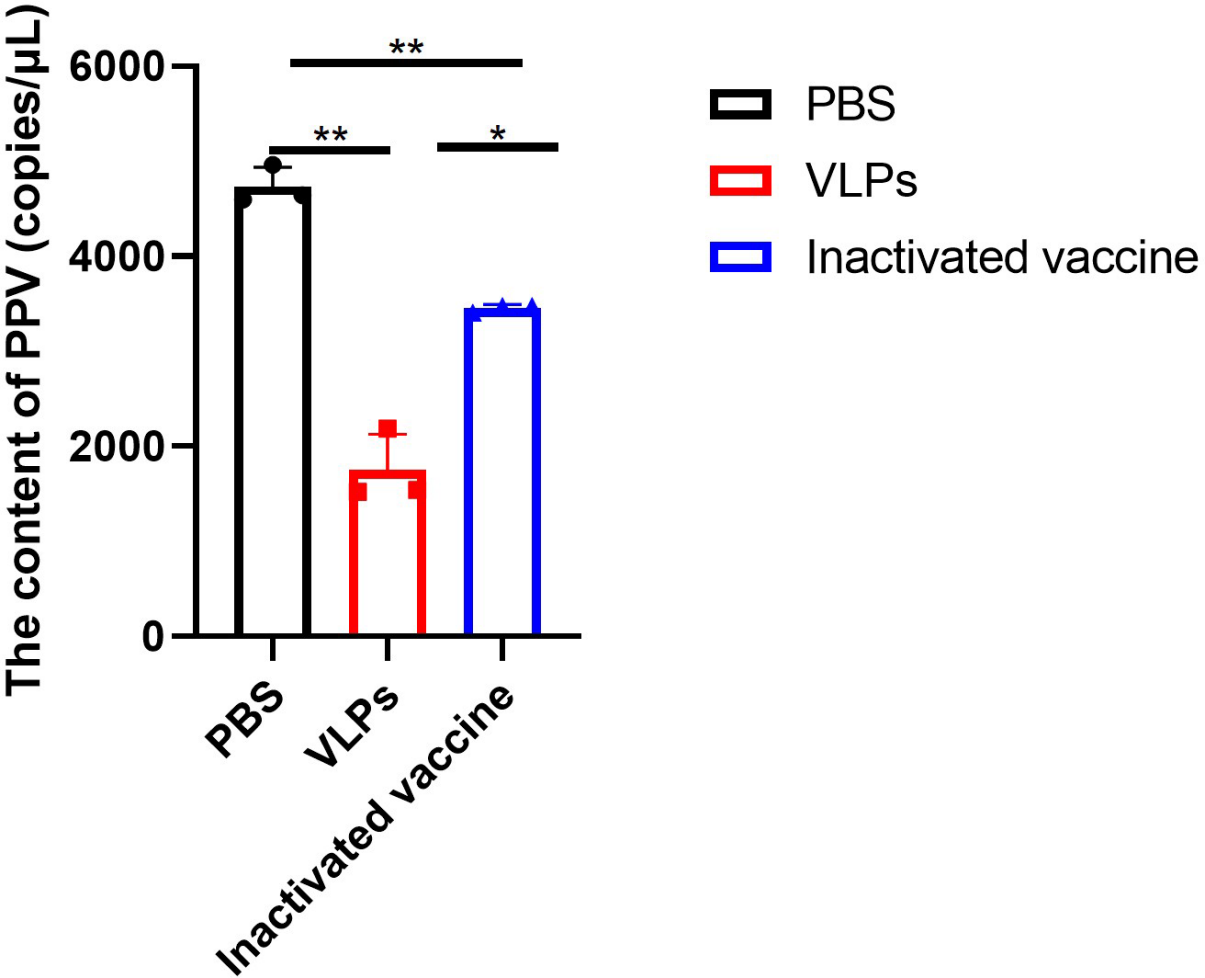
Quantification of viral load in mouse spleens by absolute qPCR. Day 14 post-challenge, total RNA was extracted from mouse tissues and subjected to absolute quantitative PCR analysis. Significance is indicated in the figure by asterisks (*, P < 0.05; **, P < 0.01).

The results demonstrated that the splenic viral load was inversely correlated with the pre-challenge HI antibody titers. Specifically, the PBS control group exhibited significantly higher viral gene copy numbers compared to both the VLP and inactivated vaccine groups (**Figure 9**). Notably, the viral load in the VLP-immunized group was significantly lower than that in the inactivated vaccine group, indicating that the VLP vaccine provides superior immunoprotection and more effective viral clearance.

## 4 Discussion

Vaccination is one of the most effective and cost-efficient strategies for PPV infection (39). Protection against PPV is primarily mediated by humoral immunity, which relies on the differentiation and maintenance of LLPCs and MBCs (12). The germinal center (GC) response serves as a critical selection phase where B cells undergo affinity maturation and differentiate into LLPCs and MBCs, making it a cornerstone for antiviral vaccine development (40) Therefore, elucidating the mechanisms by which PPV vaccines activate the GC reaction is of significant importance for enhancing vaccine-induced humoral immune responses.

Commercially available PPV vaccines are predominantly based on chemically inactivated whole-virus formulations, which have been widely used in swine production due to their safety and established efficacy in preventing reproductive failure. However, accumulating evidence suggests that such vaccines may exhibit limitations in inducing long-lasting immunity and broad protection against emerging variant strains. For instance, antigenic drift in PPV capsid proteins has been reported to reduce the cross-reactivity of vaccine-induced antibodies against contemporary circulating strains (1). In contrast, VLP-based vaccines, which structurally mimic native virions while lacking viral genetic material, have demonstrated enhanced immunogenicity and safety profiles. Previous studies have shown that PPV VLPs can induce stronger humoral immune responses and higher neutralizing antibody titers compared to inactivated vaccines (12). This advantage is generally attributed to their highly repetitive and particulate structure, which facilitates efficient uptake by antigen-presenting cells and promotes robust germinal center reactions. Moreover, VLP-based platforms have been reported to induce more durable immune responses by enhancing the generation of long-lived plasma cells and memory B cells, thereby contributing to sustained antibody production (38). These features are particularly relevant in the context of PPV, where long-term protection is critical for preventing transplacental infection.

Subunit vaccines derived from prokaryotic expression systems offer economic advantages and play a vital role in controlling porcine diseases (12). Recently, virus-like particles (VLPs), which closely mimic the quaternary structure of native viruses without being infectious, have emerged as a potent vaccine platform (41). In this study, we produced PPV VLPs using an *E. coli* expression system optimized with molecular chaperones. The resulting VLPs exhibited authentic morphological features, retained hemagglutination activity, and elicited robust GC and Tfh cell responses, leading to durable protective antibody titers. Collectively, these findings highlight the potential of these VP2 VLPs as promising candidates for next-generation PPV vaccines.

A key finding of this study is that PPV VLPs induced significantly stronger PPV-specific IgG responses and higher HI titers compared to inactivated vaccines. This aligns with observations in other VLP platforms, such as MERS-CoV VLPs (42), which elicit superior protective humoral immunity The high HI titers indicate that our PPV VLPs can induce potent neutralizing antibody responses. VLPs have consistently outperformed soluble protein vaccines in terms of the durability and breadth of immunity, particularly when formulated with modern adjuvants like ISA 201 VG (43, 44). Furthermore, the VLP vaccine induced robust CD4^+^ T-cell responses, characterized by increased secretion of IL-4, IL-6, IFN-γ, and TNF-α, suggesting a balanced Th1/Th2 activation. Similar patterns have been reported for hepatitis E and AAV VP3-only VLPs produced in *E. coli*, both of which elicited strong cellular responses and antigen-specific T-cell activation (45, 46).

Most importantly, our data demonstrate that PPV VLPs promote potent GC and Tfh cell activation. Sustained GC activity is a hallmark of high-quality humoral immunity, enabling the generation of high-affinity LLPCs. Recent studies on SARS-CoV-2 mRNA vaccines (47, 48) have highlighted that prolonged GC reactions can persist for months, and nanoparticle/VLP-based vaccines are recognized as particularly effective inducers of this pathway (49). Our observation of expanded GC B cells, Tfh cells, and LLPCs, coupled with the upregulated expression of *Il21*, *Bcl2*, and *Prdm1* (Blimp-1), strongly suggests that PPV VLPs stimulate this high-fidelity immune axis more effectively than traditional inactivated vaccines. Given the structural plasticity of PPV VP2, future work could leverage this VLP as a multivalent platform to present epitopes from other pathogens, such as FMDV (50).

PPV VLP vaccination reshaped the lymphoid immune landscape at single-cell resolution by selectively expanding germinal center-associated B-cell subsets, plasma cells, and helper CD4^+^ T-cell populations. Transcriptomic profiling revealed that GC B cells in the VLP group displayed strong proliferative and activation signatures, with upregulation of key cell-cycle and GC-associated genes, consistent with enhanced clonal expansion and affinity maturation. Differential expression analysis and enrichment analysis further confirmed that VLP immunization activates the core immunoregulatory pathways of humoral immunity, including BCR/TCR signaling, antigen processing and presentation, and the TNF signaling pathway, with the BCR signaling pathway being particularly prominent. The first signal for B cell activation is initiated by the specific recognition and binding of vaccine antigens (such as proteins or polysaccharides) to the B cell surface BCR. The core mechanism is as follows: cross-linking of the BCR activates Syk kinase, which subsequently triggers the downstream PI3K/Akt and MAPK/ERK pathways, promoting B cell proliferation and survival. At the same time, BCR signaling also activates the NF-κB pathway via the CARD11/BCL10/MALT1 complex, inducing the expression of genes related to B cell activation. This signal is crucial for determining whether B cells can be activated by specific antigens and is an important prerequisite for B cells to enter the germinal center reaction.Together, these findings indicate that PPV VLPs promote coordinated activation of the GC-Tfh-plasma cell network, providing a mechanistic basis for the improved antibody maturation and durable humoral protection elicited by VLP vaccination.

Notably, this study has three key limitations to be addressed: first, in the present study, PPV VLPs were formulated with the ISA 201 VG adjuvant, whereas the adjuvant composition of the commercial inactivated vaccine was not explicitly defined. Moreover, the absence of critical control groups, including an adjuvant-only group and a non-adjuvanted VLP group, prevents a clear dissection of the respective contributions of antigen structure and adjuvant effect. Given that ISA 201 VG is a potent water-in-oil-in-water (W/O/W) emulsion capable of inducing strong humoral immune responses (23), the enhanced antibody production and germinal center reactions observed in the VLP group may partially result from adjuvant-mediated immunostimulation rather than solely the intrinsic properties of the VLPs. Therefore, it remains difficult to conclusively attribute the observed immunological advantages to the VLP platform itself. Future studies should incorporate more rigorous control designs, including VLPs with and without adjuvant, adjuvant-only controls, and vaccines formulated with standardized adjuvant systems across groups. Such designs would allow for decoupling of antigen-specific effects from adjuvant-driven responses and enable a more precise evaluation of the intrinsic immunogenicity of PPV VLPs. Second, mice are not the natural host of PPV, and the protective efficacy in the mouse challenge model needs further validation in relevant surrogate models and swine. Guinea pigs have been well-established as a reliable animal model for PPV vaccine evaluation, showing consistent antibody responses to PPV attenuated and inactivated vaccines (26, 51), and comparative analyses have confirmed analogous PPV vaccine-induced antibody profiles between guinea pigs, rabbits and pigs. Future studies are therefore required to validate the VLP vaccine’s immunogenicity and protection in guinea pigs and the natural swine host to strengthen the translational relevance of our findings. A further limitation of this study is that single-cell RNA sequencing (scRNA-seq) analysis was performed only on the VLP-immunized group and the PBS control group, without including the commercial inactivated vaccine group. Therefore, a direct head-to-head comparison of the transcriptomic profiles between the VLP and inactivated vaccine groups at the single-cell level is lacking. This precludes us from definitively determining which upregulated signaling pathways and transcriptional programs are uniquely associated with VLP vaccination.

In conclusion, our results demonstrate that PPV VP2 VLPs produced in *E. coli* represent a promising next-generation vaccine candidate with advantages in immunogenicity, scalability, safety, and durability. Future studies should focus on challenge experiments in the target host (swine), evaluation of cross-strain protection, and the exploration of multivalent VLP platforms. The growing body of evidence from recent VLP research supports the transition of this platform toward practical application in swine health management.

## Data Availability

The data presented in the study are deposited in the NCBI repository, accession number PRJNA1464174.
