# Supplementary material for: Porcine Parvovirus virus-like particle vaccine induces long-term humoral immunity by recruiting T follicular helper and germinal center B cell responses
Source: Front Immunol. 2026 May 26;17:1817304. doi: 10.3389/fimmu.2026.1817304 (PMC13246690; doi:10.3389/fimmu.2026.1817304)
Supplement: Supplementary file 1 [file DataSheet1.pdf]

Supplementary Material

Porcine Parvovirus virus-like particle vaccine induces long-term humoral immunity by recruiting T follicular helper and germinal center B cell responses

1. Supplementary Figures

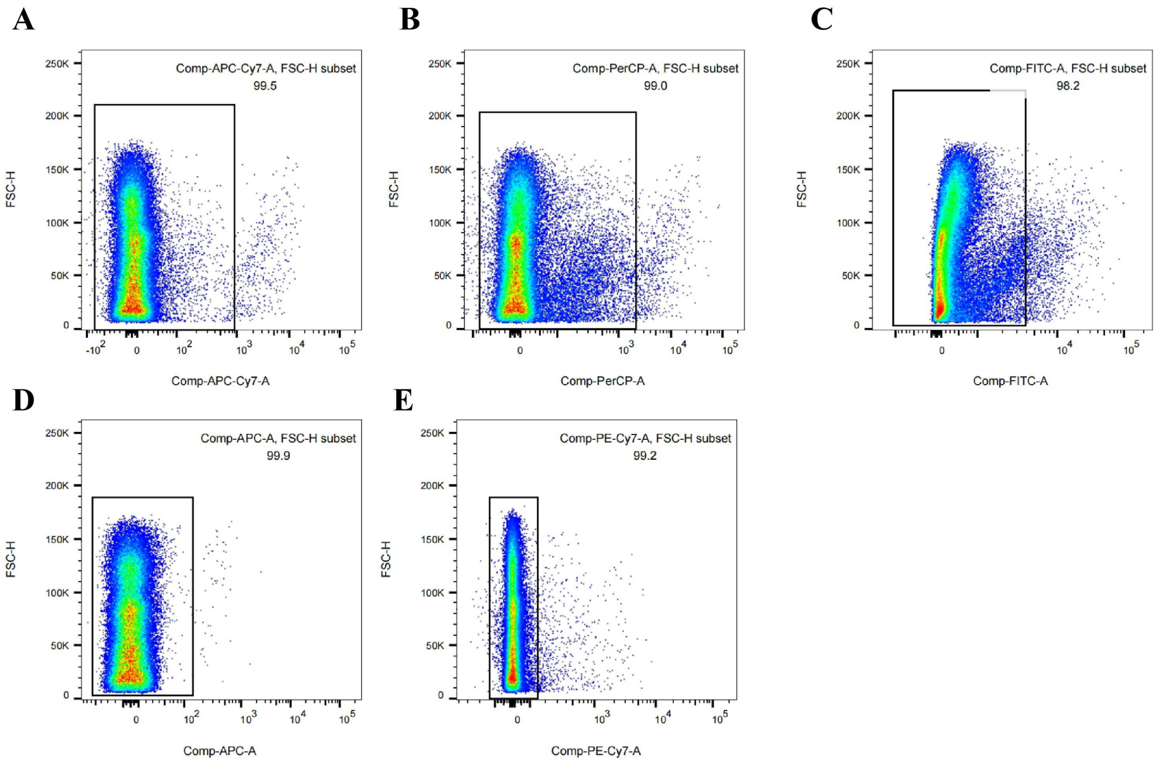

Supplementary Figure 1. Five-color flow cytometry FMO controls, with FSC-H on the x-axis and the signal intensity of the corresponding fluorescence channel on the y-axis. (A) Fluorescence minus one (APC-Cy7) channel negative cell percentage; (B) Fluorescence minus one (PerCP) channel negative cell percentage; (C) Fluorescence minus one (FITC) channel negative cell percentage; (D) Fluorescence minus one (APC) channel negative cell percentage; (E) Fluorescence minus one (PE-Cy7) channel negative cell percentage.

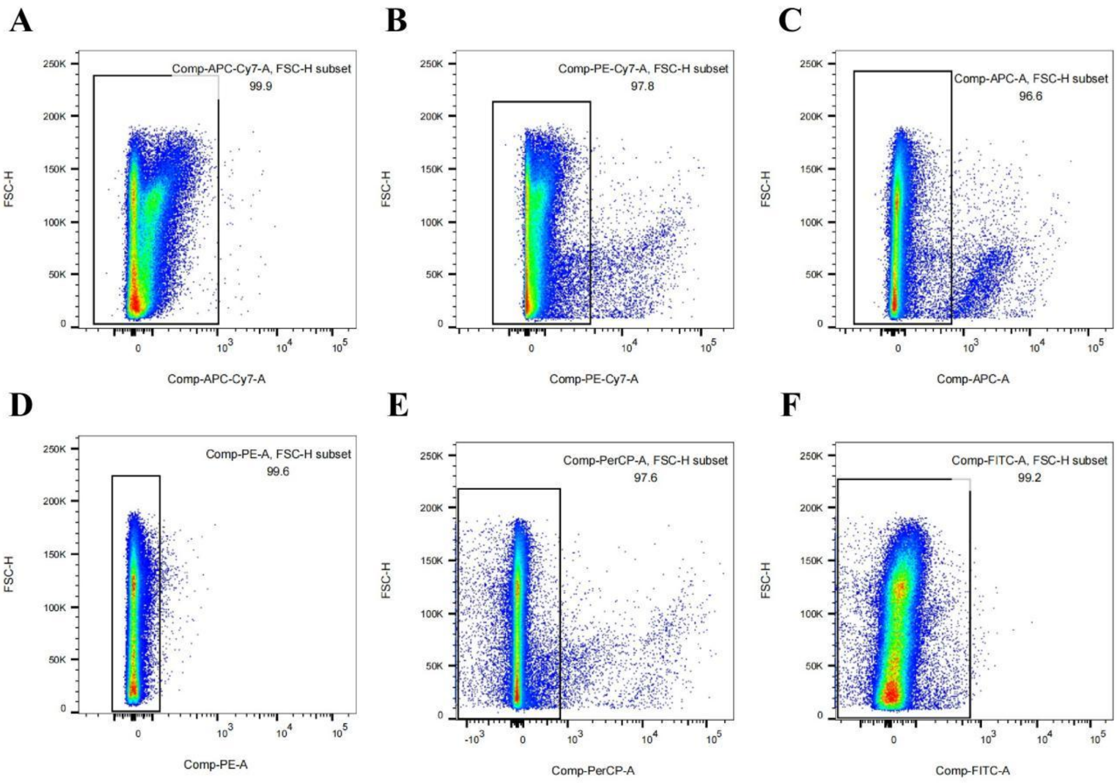

Supplementary Figure 2. Six-color flow cytometry FMO controls, with FSC-H on the x-axis and the signal intensity of the corresponding fluorescence channel on the y-axis. (A) Fluorescence minus one (APC-Cy7) channel negative cell percentage; (B) Fluorescence minus one (PE-Cy7) channel negative cell percentage; (C) Fluorescence minus one (APC) channel negative cell percentage; (D) Fluorescence minus one (PE) channel negative cell percentage; (E) Fluorescence minus one (PerCP) channel negative cell percentage; (F) Fluorescence minus one (FITC) channel negative cell percentage.
